# Unilateral breast Darier disease: A case report and literature review

**DOI:** 10.3892/mi.2025.244

**Published:** 2025-05-26

**Authors:** Lana R.A. Pshtiwan, Shaban Latif Tofiq, Dilan S. Hiwa, Harzal Hiwa Fatih, Karukh K. Mohammed, Abdulwahid M. Salih, Diyar A. Omar, Kayhan A. Najar, Fahmi H. Kakamad

**Affiliations:** 1 Department of Scientific Affairs, Smart Health Tower, Sulaymaniyah 46001, Iraq; 2 Department of Radiology, Shar Teaching Hospital, Sulaymaniyah 46001, Iraq; 3 Smart Health Tower (Raparin Branch), Ranya, Sulaymaniyah 46001, Iraq; 4 Kscien Organization for Scientific Research (Middle East office), Sulaymaniyah 46001, Iraq; 5 College of Medicine, University of Sulaimani, Sulaymaniyah 46001, Iraq; 6 Department of Medical Laboratory Technology, Shaqlawa Technical College, Erbil Polytechnic University, Erbil 44001, Iraq

**Keywords:** keratosis follicularis, localized Darier disease, Darier-White disease, areola, mammary gland, nipple

## Abstract

Darier disease is often an underrecognized, disfiguring skin condition. Although the localized form of this condition has been reported in several parts of the body, the present study describes the case of a patient with the unique involvement of one breast, destroying the nipple. A 35-year-old female patient complained of having a right nipple lesion for 1 month. There were multiple small brown-to-black firm nipple lesions causing nipple destruction, associated with multiple small red, non-tender skin lesions in the areolar region and lower part of the breast. An incisional biopsy revealed a small number of acantholytic cells and a papillary-like configuration within the epidermis; no conclusive evidence of malignancy was observed, and a diagnosis of Darier disease was made. Emollient lotion, topical fusidic acid cream 2%, and oral isotretinoin 20 mg once daily were prescribed. The proposed pathogenesis of localized Darier disease is due to genetic mosaicism; these mutations arise during zygotic division. It can present as a rash with either macular or linear patterns confined to a specific body area. In the literature review, localized Darier disease affecting the inframammary region, vulva, axilla, and side of the abdomen have been reported, among others. Paget's disease was hidden in the background of Darier's disease of the breast in one of the cases in the literature. Darier disease may present as a localized unilateral breast lesion, characterized by multiple small, firm brown to black lesions on the nipple, leading to nipple destruction.

## Introduction

Darier disease is a rare, often misdiagnosed genetic disorder inherited in an autosomal dominant pattern. It was initially described by Darier and White in 1889. Sporadic cases are estimated to occur in approximately half of the instances, with the condition exhibiting high penetrance, >95%. Darier disease typically begins in childhood and continues into adolescence, leading to the formation of small papules, primarily in the seborrheic regions, including the scalp, the areas behind the ears and temples, the back of the neck, the front of the torso and skin folds. Additionally, the involvement of the palms, soles, nails and mucous membranes may occur. Over time, these areas may become covered with scales and yellowish or brown crusts characterized by hyperkeratosis. The papules do not always develop within hair follicles, but frequently cluster together, forming wart-like lesions covered with keratotic crusts. Nonetheless, there are instances where symptoms may not appear until the sixth or seventh decade of life, with localized Darier disease being a clinical variant that was first identified by Kreibich in 1906 (1-3).

A mutation in the ATPase sarcoplasmic/endoplasmic reticulum Ca^2+^ transporting 2 (ATP2A2) gene located on chromosome 12q23-24 is attributed to the pathogenesis of Darier disease due to a malfunction of the endoplasmic reticulum Ca²^+^ ATPase pump. This malfunction leads to defective calcium storage within the aforementioned organelle. Consequently, this disruption impairs the normal processing of junctional proteins, such as desmoplakins, leading to acantholysis (4).

The present study describes a sporadic case of Darier disease localized only to the breast. The validity of the references in the present case report was confirmed, and the case report was written according to CaReL guidelines (5,6).

## Case report

### Patient information

A 35-year-old female sought medical attention at Smart Health Tower, Ranya, Iraq on August 14, 2024, due to a right nipple lesion that had persisted for approximately one month. She delivered two children via cesarean section, both of whom were breastfed for a combined duration of 3 years and 2 months. Her past medical history was unremarkable, apart from a family history of lung cancer in her paternal aunt.

### Clinical findings

**Figure 1:**
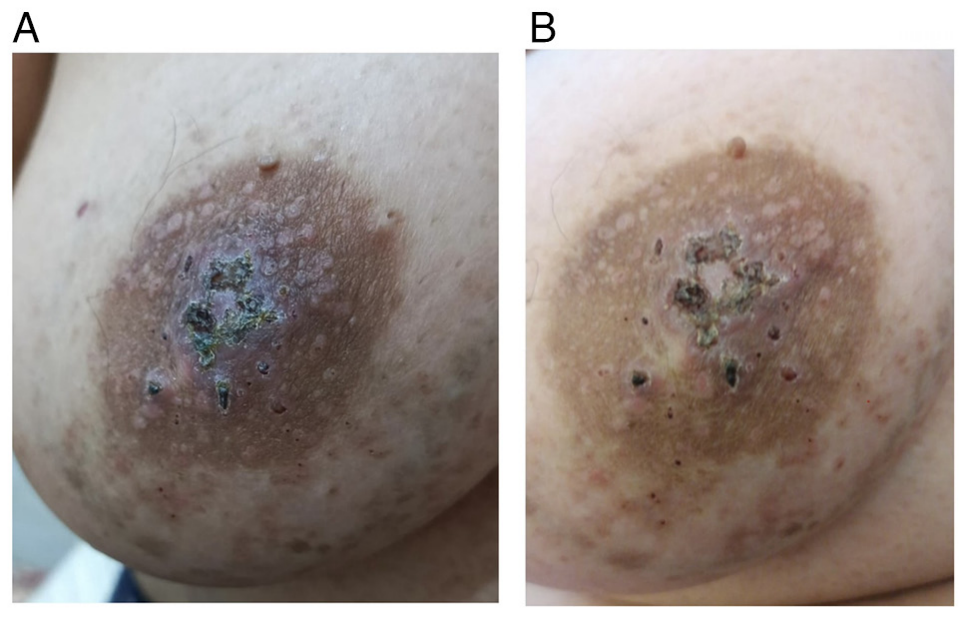
(A) Multiple small brown-to-black firm nipple lesions causing nipple destruction. (B) This was associated with multiple small red, non-tender skin lesions in the areolar region and lower part of the right breast.

There were multiple small brown to black firm nipple lesions causing nipple destruction, associated with multiple small red, non-tender skin lesions in the areolar region and the lower part of the breast (Fig. 1).

### Diagnostic approach

**Figure 2:**
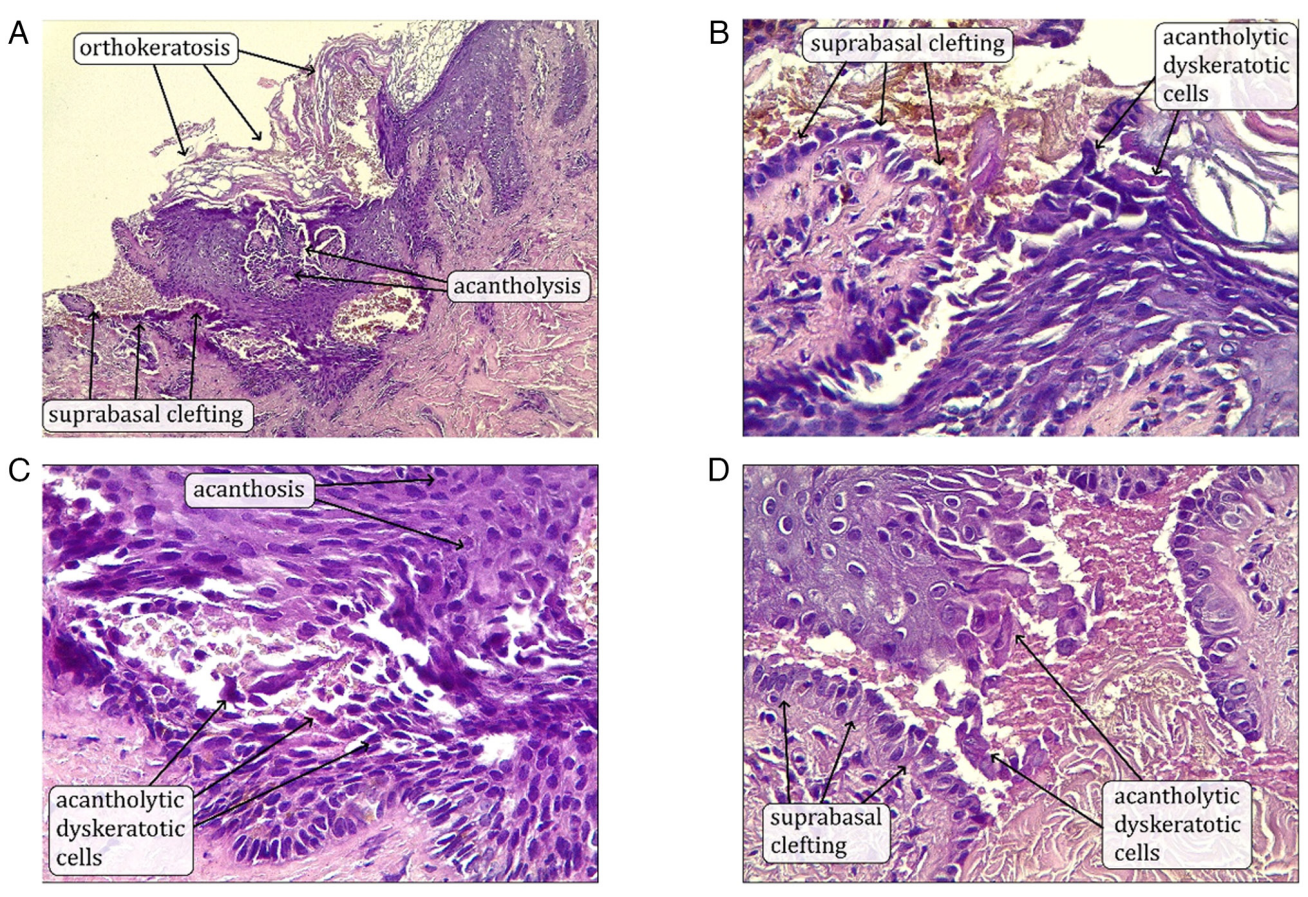
(A-D) The breast skin lesion biopsy shows mild acanthosis and orthokeratosis overlying an area of suprabasal epidermal clefting with papillary projections of the dermis and acantholytic, dyskeratotic cells at the level of the spinous layer [(A-D) Hematoxylin and eosin staining; original magnification: (A) x100, (B-D) x400].

The breast ultrasound revealed homogenous background echotexture with a fibroglandular pattern, normal morphology, and no solid masses or distortion in either breast. The breasts had normal skin thickness and contours, and non-specific axillary lymph nodes were observed. The right nipple was not visible and the ulcerated areola complex was suggestive of nipple adenoma. Tissue diagnosis was obtained from the right nipple lesion by incisional biopsy. The histopathological examination was performed in the following manner; The sections used were paraffin-embedded and sectioned to a thickness of 5 µm. Fixation was performed using 10% neutral-buffered formalin at room temperature for 24 h. Hematoxylin and eosin (H&E) staining (from Bio Optica) was used with hematoxylin staining carried out for 5 min and eosin staining for 2 min, both at room temperature. The slides were observed and imaged using a light microscope (Leica Microsystems GmbH). The results revealed a small amount of acantholytic cells and papillary-like configuration within the epidermis; no conclusive evidence of malignancy was observed, and a diagnosis of Darier disease localized to the breast was made (Fig. 2). The patient was advised to undergo ATP2A2 gene analysis to support the diagnosis; however, the test was not performed.

### Therapeutic intervention

The patient was administered high-dose oral Costus medication to reduce inflammation at a dose of three capsules (each containing 250 mg Costus extract) taken twice daily, totaling 1,500 mg per day. This treatment was maintained for a period of 6 months, after which the dose was gradually tapered based on the clinical response and tolerance of the patient. Her dermatologist recommended emollient lotion to relax and hydrate her skin, topical fusidic acid cream 2% to control and prevent further skin infection, and oral isotretinoin 20 mg once daily to minimize hyperkeratosis and smoothen papules. The patient was instructed to wear sunscreen and avoid sun exposure. For monitoring clinical improvement, follow-up appointments were planned on a regular schedule.

### Follow-up

The patient is still undergoing regular checkups and is in good health without any health issues. Although routine baseline and follow-up laboratory tests, including liver function tests (LFTs) and lipid profile were recommended, the patient declined to proceed with these investigations. Following 3 months of isotretinoin use, the medication was gradually tapered and discontinued due to the desire of the patient to conceive in the following 6 months. Furthermore, the teratogenic risks associated with the use of isotretinoin were clearly explained, and appropriate pregnancy prevention counseling was provided. Currently, the condition of the patient remains stable, with no signs of progression or new complications.

## Discussion

Darier disease is a hereditary condition characterized by abnormal keratinization of the skin. Apart from skin manifestations, it may also manifest with non-dermal symptoms, including psychiatric conditions, such as intellectual disability, epilepsy, or bipolar disorder (1). However, the patient in the present study did not complain of any psychiatric issues, and there were no such complaints in the review of the literature as regards localized drier disease, apart from a 5-year-old female with Darier disease localized to the vulva; however, it is worth mentioning that the epilepsy and mental impairment she was suffering from were attributed to a cardiopulmonary arrest event earlier in her life rather than Darier disease itself (7). The outcomes of localized Darier disease across the referenced studies were generally favorable with either complete resolution or significant improvement, particularly when appropriate topical treatments were applied. However, one case did report relapse following treatment discontinuation (Table I) (2,3,7-9). This can be explained by the proposed pathogenesis of localized Darrier disease, which is due to genetic mosaicism, as previously mentioned by Takagi *et al* (1); these mutations arise during zygotic division. It presents as a rash with either macular or linear patterns, confined to a specific body area, resembling an epidermal nevus distribution (1). Only a limited number of confirmed cases of localized Darier disease have been reported, at least to the best of our knowledge. For instance, Fitzgerald and Lewis-Jones (2) reported the case of a 59-year-old female patient who presented with plaque over each areola consisting of numerous crusted, brownish papules, which were confirmed as Darier disease on histopathological examination. When it is confined to the areola with no other parts of the body affected, it can easily be misdiagnosed, as was the case with the patient in the present study; this was only diagnosed following a histopathological examination. The opposite was reported by Spizuoco *et al* (4), where a patient presented with widespread, merging, keratotic, crusted, and papular lesions located in the scalp, forehead, back and chest, in addition to her left nipple being affected. She was diagnosed with Darier disease; however, later on, a biopsy of the nipple lesion revealed confirmatory findings of Paget's disease, necessitating a detailed approach and considering possible differential diagnosis (4). The other differentials include Hailey-Hailey disease, Paget's disease, Seborrheic dermatitis, epidermodysplasia verruciformis and acanthosis nigricans necessitating histopathological examination for confirming query cases, which shows the stratum corneum with disorganized hyperproliferation and keratotic plug formation, accompanied by the presence of parakeratosis. In regions where suprabasal cleavage occurs, there is a notable presence of acantholytic cells along with atypical keratinocytes, which manifest as corps ronds and grains. These pathological features are distributed throughout the affected tissue, reflecting disruptions in cellular adhesion and differentiation (1). The case in the present study underwent breast ultrasound to detect any underlying mass or tumor, as it is important to exclude breast cancer in such cases as breast cancer is one of the leading causes of mortality and morbidity worldwide (10).

Currently, no validated curative treatments exist; the majority of cases are managed symptomatically. Providing lifestyle guidance is crucial in mitigating exacerbating factors, including high humidity, elevated temperatures, excessive sweating, mechanical irritation, exposure to ultraviolet rays, pregnancy, childbirth, and surgical interventions. In the present case, 20 mg of daily isotretinoin was used, which is frequently employed in treating Darier disease and is highly effective. However, the use of this drug is associated with several side-effects; thus, a number of patients undergo only intermittent therapy or, in some cases, discontinue treatment altogether, particularly for females wishing to conceive (1).

Additionally, *in vitro* studies have demonstrated that treating cultured keratinocytes with the three medications prednisolone, cyclosporine, and retinoids can alleviate the suppression of ATP2A2 gene expression following ultraviolet irradiation (1,11). This suggests the potential therapeutic efficacy of these drugs in managing the condition. However, in instances where Darier disease is localized, topical options can offer fewer side-effects with similar outcomes; for example, in their study, Linder *et al* (3) used 0.1% adapalene cream for Darier disease localized to the inflammatory region, which resulted in complete resolution of the skin lesions. Furthermore, O'Malley *et al* (9) reported similar results with topical tretinoin, resulting in a complete resolution in 4 months involving the right axilla in one of their four cases. In another case reported by Salopek *et al* (7), antifungal treatment and corticosteroids were used without notable efficacy; however, later on, a barrier cream resulted in a good outcome. Observation or treatment with mild corticosteroid and barrier creams can be considered for localized forms of Darier disease (1,3,7,9).

The present study had certain limitations which should be mentioned. A limitation of the present study is the unavailability of ultrasound images, laboratory tests (including LFTs and lipid profile tests), ATP2A2 gene analysis, and other supporting investigations to confirm the underlying cause of Darier disease.

In conclusion, Darier disease may present as a localized unilateral breast lesion, characterized by multiple small, firm brown to black lesions on the nipple, leading to nipple destruction.

## Figures and Tables

**Table I:** Characteristics of the patients with localized Darier disease identified in the literature.

First author, year of publication	No. of patients	Sex	Age (years)	Family history of Darier disease	Localization	Presentation	Comorbidities	Treatment	Outcome	Follow-up (months)	(Refs.)
Fitzgerald, 1997	1	F	59	No	Both breasts	Plaque over each areola consisting of numerous crusted, brownish papules.	Asthma, angina, hypothyroidism and cervical spondylosis	-	Significant improvement, but appearance of multiple small palmar pits and V-shaped notching of the distal ends of several of the nails	Unspecified	(2)
Salopek, 1993	1	F	5	No	Vulva	Red-brown, 1-2-mm papules coalesced to form confluent plaques over the vulvar and perivulvar skin in a bilateral, symmetric distribution. The surface of the papules was rough and somewhat warty, with a few lesions revealing superficial erosions. In the left groin was an ulcerated, crusted papule.	Mentally impaired and suffered from epilepsy secondary to aspiration-induced cardiopulmonary arrest at age ten months	Antifungals for possible candidiasis, and corticosteroids for diaper dermatitis, without much success. Barrier cream was lastly used.	Spontaneous resolution	3	(7)
Barrett, 1989	1	F	43	No	Vulva	Pruritic lesion.	-	Mild corticosteroid cream	Complete resolution	3	(8)
Linder, 2016	1	F	71	No	Inframammary and presternal area	Several symmetrically distributed, small, very itchy, partly excoriated red papules surrounded by an erythematous halo. The lesions were 4-8 mm in diameter.	-	Daily 0.1% adapalene cream	Complete resolution	Unspecified	(3)
O'Malley, 1997	4	M	21	No	Right side of the chest	Asymptomatic linear group of yellow-brown keratotic papules for five years.	-	No treatment received	-	-	(9)
		M	37	No	Left side of the abdomen	Linear group of discrete, warty, brown papules.	-	0.1% tretinoin cream	Complete resolution	4	
		M	36	No	Right axilla	Brown, keratotic papules anteriorly and a macerated, vegetative, erythematous plaque.	-	-	-	-	
		F	35	No	Posterior aspect of right leg, right arm and the right side of trunk.	Rough, yellowish papules .	-	A 2-year course of treatment with a combination of vitamin A cream and betamethasone valerate	The eruption cleared, but it gradually returned after the treatment was discontinued	24	

M, male; F, female.

## Data Availability

The data generated in the present study may be requested from the corresponding author.
